# Visual performance following implantation of presbyopia correcting intraocular lenses

**DOI:** 10.1038/s41433-022-02188-y

**Published:** 2022-08-08

**Authors:** Magda A. Torky, Amgad El Nokrashy, Heba Metwally, Ameera G. Abdelhameed

**Affiliations:** 1 Department of Ophthalmology, Mansoura Ophthalmic Center, Faculty of Medicine, Mansoura University, Mansoura, Egypt; 2 Memorial Institute of Ophthalmic Research, Mansoura, Egypt

**Keywords:** Medical research, Health care

## Abstract

**Objective:**

To compare the visual outcomes following bilateral implantation of 3 presbyopia correcting IOLs.

**Methods:**

Results are reported for patients who underwent phacoemulsification with bilateral implantation of one of the following IOLs: Panoptix IOL, AT LISA tri IOL and Symphony IOL. Six months postoperative examination included monocular UIVA at 80 and 60 cm and monocular UNVA and DCNVA at 40 cm, monocular UDVA and CDVA, Spherical equivalent (SE) refraction, binocular defocus curve, contrast sensitivity, photopic phenomena, and spectacle independence.

**Results:**

UIVA at 80 cm was significantly better in the AT LISA group and the Symfony groups than the Panoptix group, while UIVA at 60 cm was better in the Symfony group and the Panoptix group than the AT LISA group. A worse DCNVA was recorded in the Symfony group. At a defocus of −2.50 D, the near VA was similar in the PanOptix and AT LISA groups (0.05, 0.07 logMAR respectively), which were significantly better than the Symfony group (0.3 LogMAR). Binocular photopic and scotopic contrast sensitivity outcomes were similar among the three groups at all spatial frequencies. In Symphony group, the frequency and the degree of bother of photic phenomena was relatively higher than the other two groups. Higher percentage of patients in symphony group reported their need for reading glasses.

**Conclusion:**

PanOptix IOL and AT LISA IOL would be a good choice for patients aiming for an optimum near vision, while Symfony IOL seems suitable for patients having the priority for good intermediate vision.

## Introduction

Multifocal intraocular lenses (MIOLs) are being increasingly implanted after cataract surgery to enhance spectacle independence [1–3]. They were initially bifocal, providing near and far foci. However, this was not totally convenient for patients requiring a sharp intermediate focus, for example computers, tablets, and handheld devices users [4]. This urged the introduction of different MIOLs to meet the growing patients’ functional vision needs [5]. Among those are the Panfocal IOL (PanOptix™ Alcon Laboratories Inc., Fort Worth, USA) [6] and AT LISA tri 839MP (Carl Zeiss Meditec AG) [7]. Both are trifocal diffractive IOLs which split light into three foci in both narrow and wide pupil conditions. They have been reported to provide accepted visual acuity (VA) for distance, intermediate and near [6–8].

More recently, a new IOL design—the Tecnis Symfony (Johnson & Johnson Surgical Vision Inc., Santa Ana, USA)—has been introduced. It is based on creation of an elongated focal point to extend the depth of focus, offering a wide range of vision, and minimizing the visual phenomena linked to multiple focal points associated with conventional MIOLs [9–12]. It is claimed to have less photic phenomena and better intermediate vision than trifocal IOLs. Therefore, it has been recommended for active lifestyle patients, who aim for spectacle independence, but are sensitive to halo and glare [9].

The current study aimed to compare the visual outcomes following bilateral implantation of 3 presbyopia-correcting IOLs, the PanOptix IOL, the AT LISA tri 839MP IOL and the Tecnis Symfony IOL.

## Patients and methods

### Study design

This was a prospective randomized clinical trial conducted at the Department of Ophthalmology, Dar Alshifa hospital, Kuwait during the period from June 2019 through May 2020. The study was approved by Dar Alshifa hospital Ethics committee. All patients signed a written consent after explanation of the surgical procedure and vision concerns of presbyopia-correcting IOLs. The study followed the tenets of the Declaration of Helsinki and adhered to the CONSORT guidelines for reporting clinical trials:and was registered on www.clinicaltrials.gov: Clinicaltrial.gov ID: NCT04907955. Unique Protocol ID: 02282021065727, https://clinicaltrials.gov/ct2/show/NCT04907955.

### Inclusion/exclusion criteria

Patients diagnosed with bilateral senile cataract, motivated for spectacle independence but with tolerance of imprecise vision, with scotopic pupil size <6 mm, and with preoperative regular corneal astigmatism below 1.0 D, were included.

The exclusion criteria included pseudoexfoliation, traumatic cataract, history of ocular surgery, glaucoma, low endothelial cell count <2000 cells/mm^2^, high myopia (axial length >25.5 mm) and hyperopia (axial length <21.5 mm), old age (>70 years) due to probable difficult neuroadaptation to the new optical conditions, history of stroke or dyslexia, unrealistic visual expectations, patients needing precise vision, for example pilots, drivers, etc., patients satisfied with reading glasses, patients unsatisfied with progressive add lenses.

### Randomization and masking

Patients were randomly (https:// www.randomizer.org) distributed for bilateral implantation of one of three non- toric presbyopia-correcting IOLs; the Acrysof IQ PanOptix TNFT00 (Alcon laboratories, Inc., Forth Worth, USA) (Group A), the AT LISA tri 839MP (Calr Zeiss MEditech, Germany) (Group B), and the TECNIS Symfony ZXR00 (J&J Vision, Inc., Santa Ana,USA) (Group C). The study was double masked. Patients were masked to the type of IOL implanted. All preoperative and postoperative assessments were done by the same author, who was masked to the type of implanted IOL.

### Intraocular lens criteria

#### Acrysof PanOptix

It is a single-piece aspheric (negative asphericity of −0.10 μm) IOL, with non-apodized diffractive design. It has a central 4.5 mm portion with 15 diffractive zones. It splits light into three foci, distance, intermediate (60 cm) and near (40 cm). The lens has an overall diameter of 13.0 mm, and an optical diameter of 6.0 mm and is available in powers from +13.0 D to +34.0 D [3, 13].

#### AT LISA tri 839MP

It is a preloaded single-piece hydrophilic acrylic IOL with a hydrophobic surface and an ultraviolet absorber. It has an aspheric diffractive design that compensates for corneal spherical aberrations. It has a central 4.34 mm trifocal zone and a peripheral 4.34–6.00 mm bifocal zone. Light is asymmetrically distributed between three foci; far (50%), intermediate (20%) and near (30%). The IOL has a near addition (add) of +3.33 D and an intermediate add of +1.66 D at 80 cm. The overall length is 11.0 mm with a 6.0 mm optical diameter with dioptric power from 0.0 to +32.0 D in 0.5 D increments [14].

#### TECNIS Symfony ZXR00

It is a hydrophobic acrylic aspheric biconvex IOL. It has a wavefront-designed anterior aspheric surface (negative spherical aberration of −0.27 µm) which compensates for the corneal net positive spherical aberrations thus improving the contrast sensitivity. The echelette design of the achromatic diffractive posterior surface elongates the depth of focus and increases the range of vision rather than splitting light into foci. This eliminates the halo effect generated by overlapping the near and far images, formed by the multifocal IOLs. The IOL has an overall diameter of 13.0 mm with an optical diameter of 6.0 mm. It has an intermediate power add of +1.75 D and the powers available range from +5.0 to +34.0 D in 0.5 D increments [11, 15].

### Preoperative evaluation

All patients had full ophthalmologic examination. This included measurement of corrected distant visual acuity (CDVA) using Early Treatment Diabetic Retinopathy Study (ETDRS) charts (ETDRS Standardized Viewer Model No. ESV 3000) at 4 m with 100% contrast under photopic conditions (85 candelas/m2), then the results were converted into logarithm of the minimum angle of resolution (logMAR) for analysis. Manifest refraction was done using Topcon C5000 Digital Eye Exam system (Topcon, Canada) to provide sphere, cylinder, and manifest refractive spherical equivalent (MRSE). Examination also included slit lamp evaluation, Goldmann applanation tonometry, fundoscopy, keratometry, corneal tomography (Sirius, CSO, Italy), and optical biometry (IOLMaster 500, Carl Zeiss Meditec AG). Intraocular In the bag IOL power calculation was done using the SRK/T formula (for AL >22.0 mm) or Hoffer Q formulas (for AL <22.0 mm). For the PanOptix and Symfony IOLs, an optimized A-constants of 119.1 was used, while for the AT LISA tri 839MP it was 118.9. Postoperative emmetropia was targeted in the three IOL groups.

### Surgical technique and postoperative care

All surgeries were performed by an experienced surgeon (YAA). All eyes had a standard 2.2 mm clear corneal incision phacoemulsification procedure with in the bag IOL implantation. Postoperative regimen included moxifloxacin 0.5%, prednisolone acetate 1% ophthalmic suspension and nepafenac 0.1% ophthalmic suspension, every 4 h for 2 weeks, then reduced gradually over 3 weeks. In all patients, the fellow eye was operated 2 weeks after the first eye with implantation of the same IOL in both eyes.

### Postoperative assessment

All patients were examined on the first day, first week, first, and sixth months postoperatively.

#### Visual acuity

The following visual acuities were assessed; monocular uncorrected distance visual acuity (UDVA) at 4 m, corrected distance visual acuity (CDVA) (4 m), uncorrected intermediate visual acuity at 60 cm (UIVA 60 cm) and at 80 cm (UIVA 80 cm), uncorrected near visual acuity (40 cm) (UNVA) and distance-corrected near visual acuity (DCNVA).

#### Binocular distance-corrected defocus curve

For evaluation of the range of functional vision, binocular defocus curve obtained 6 months postoperatively under photopic conditions (85 candelas/m^2^), with distance correction worn, using ETDRS charts at a distance of 4 m. Defocusing lenses from +1.00 D to −4.00 D were introduced in 0.50 D steps.

#### Contrast sensitivity

Binocular contrast sensitivity at 4 meters was measured 6 months postoperatively with spectacle correction worn if needed. The CSV-1000 system (Vector Vision Inc. Greenville, USA) was used, under photopic and mesopic conditions without glare, with spatial frequencies of 3–18 cycles/degree.

#### Photic phenomena and spectacle independence

Patients were asked to answer a questionnaire about the frequency, severity and the degree of bother of haloes, glare and starburst and another Yes/No questionnaire about spectacle independence for far, intermediate and near vision. ^16^

### Outcome measures

*Primary outcome measures included* monocular UIVA at 80 and 60 cm and monocular UNVA and DCNVA at 40 cm, 6 months after second eye surgery.

*Secondary outcome measures included* monocular UDVA and CDVA at 4 m, SE refraction, binocular distance-corrected defocus curve, contrast sensitivity, photic phenomena, and spectacle use 6 months after second eye surgery.

### Sample size determination

For sample size calculation G*power 3.1.9.2 software was used, based on the mean monocular UIVA at 80 cm, considering 0.36 LogMAR to be clinically significant difference with 0.13 as standard deviation [16]. Accordingly, for an alpha value of 0.05 and power of 0.95, the calculated minimum sample size was 105 eyes. A 20% of this number was added to compensate for the loss to follow-up (21 eyes), hence the final sample size was 126 eyes (42 eyes per group).

### Statistical analysis

Statistical analysis was performed using the Statistical Package for Social Sciences (SPSS) version 25 (version 22.0, IBM Corp.). Normality of data was checked by Kolmogorov–Smirnov test. Numbers and percentages were used to express qualitative data while means (±SD) or medians (first and third quartiles: Q1, Q3) were used to express quantitative data. Inter-group comparisons were performed using One-way ANOVA test for normally distributed data and Chi square test for binomial and ordinal data. Visual outcomes were compared between IOL groups using the Kruskal–Wallis test, with the Bonferroni adjustment for the post hoc analysis. *P* value ≤ 0.05 was considered as statistically significant one.

## Results

**Fig. 1:**
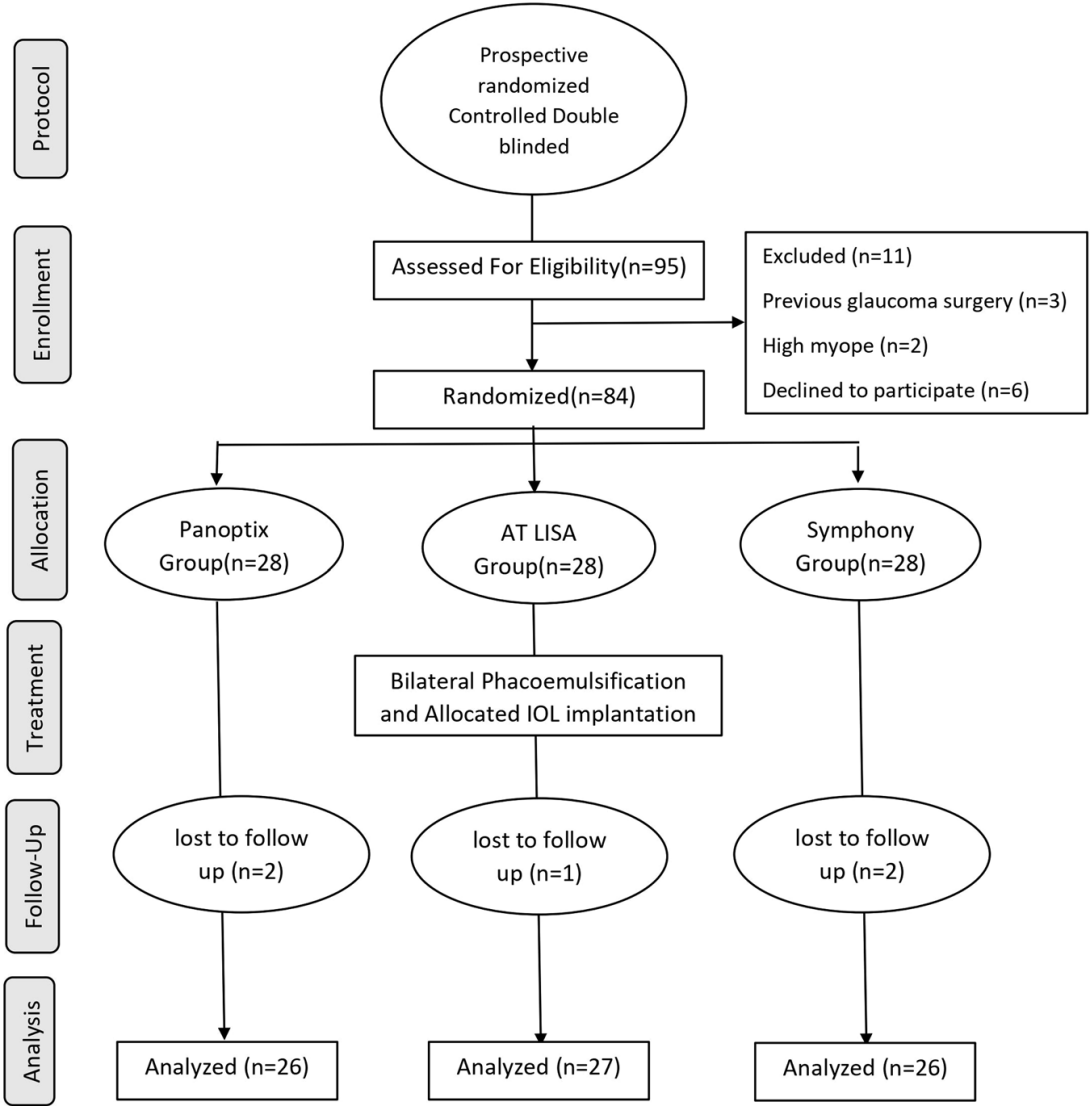
CONSORT flow chart. A diagram showing participants flow throught each satge of the study (enrollement, allocation, treatment, follow up, and analysis), n number.

Ninety-five patients were enrolled for the study. Only 84 (168 eyes) were eligible and randomized into the 3 groups, 28 patients (56 eyes) in each group. However, only 79 patients completed the 6 months follow-up: 26 patients (52 eye) in group A (Panoptix), 27 patients (54 eyes) in group B (AT LISA tri) and 26 patients (52 eye) in group C (Symfony) (Fig. 1). There were no statistically significant differences among the 3 groups in baseline characteristics (Table 1). No intraoperative or postoperative complications were encountered.

**Table 1:** Demographic characteristics of patients by group.

Characteristic	PanOptix	AT LISA	Symphony	*P*
No (patients/eyes)	26/52	27/54	26/52	
Age	59.4 ± 6.1	62.1 ± 4.2	60.5 ± 5.5	0.18^a^
	(50–69)	(52–70)	(52–69)	
Sex				0.3^b^
Male	11 (42.3%)	17 (63%)	13 (50%)	
Female	15 (57.7%)	10 (37%)	13 (50%)	
Sphere				0.9^c^
Median	1.8	2.1	−1.8	
Min, Max	(−7.0, 6.0)	(−9.0, 6.75)	(−8.0, 6.0)	
Q1, Q3	(−4.6, 3.75)	(−5.0, 3.75)	(−4.5, 3.25)	
Cylinder				0.22^c^
Median	−0.25	0.00	0.00	
Min, Max	(−1.0, 0.9)	(−0.9, 0.84)	(−1.0, 1.00)	
Q1, Q3	(−0.5, 0.25)	(−0.5, 0.34)	(−0.32, 0.45)	
SE				0.9^c^
Median	1.68	2.06	−1.69	
Min, Max	(−7.74, 6.08)	(−9.5, 6.97)	(−8.5, 6.5)	
Q1, Q3	(−4.75, 3.84)	(−5.03, 3.76)	(−4.6, 3.44)	
CDVA (logMAR)				0.43^c^
Median	0.61	0.5	0.53	
Min, Max	(0.18, 0.95)	(0.16, 0.9)	(0.18, 1.00)	
Q1, Q3	(0.48, 0.8)	(0.4, 0.71)	(0.41, 0.8)	
K1				0.56^c^
Median	7.6	7.5	7.7	
Min, Max	(7.1, 8.5)	(7.1, 8.5)	(7.2, 8.0)	
Q1, Q3	(7.4, 7.8)	(7.3, 7.8)	(7.4, 7.8)	
K2				0.6^c^
Median	7.9	7.9	7.9	
Min, Max	(7.3, 8.5)	(7.1, 8.5)	(7.6, 8.5)	
Q1, Q3	(7.7, 8.15)	(7.7, 8.15)	(7.8, 8.1)	
AL (mm)				0.57^c^
Median	22.35	22.6	23	
Min, Max	(21, 26)	(21, 29)	(21, 27)	
Q1, Q3	(21.6, 23.4)	(21.8, 23.4)	(22, 23.9)	
ACD (mm)				0.89^c^
Median	3.2	3.3	3.25	
Min, Max	(2.5, 4.2)	(2.5, 4.3)	(2.4, 4.4)	
Q1, Q3	(3.2, 3.5)	(3.17, 3.42)	(3.1, 3.5)	
IOL power(D)				0.4^c^
Median	22.0	22.0	22.0	
Min, Max	(13, 28)	(6, 27.5)	(8, 26.5)	
Q1, Q3	(21, 23)	(20, 23)	(21, 23)	

*SE* Spherical equivalent, *CDVA* Corrected distance visual acuity, *K1* steep K, *K2* Flat K, *AL* Axial length, *ACD* Anterior chamber depth, *IOL* Intraocular lens, *Q1* first quartile, *Q3* third quartile.

^a^One-way ANOVA test.

^b^Chi square test.

^c^Kruskal–Wallis.

### Visual acuity

The postoperative visual outcomes in the three studied groups were shown in Table 2. Both UDVA and CDVA were similar among the three groups (*P* = 0.23, 0.3 respectively). UIVA at 80 cm was significantly better in the AT LISA (group B) and the Symfony (group C) when compared to the Panoptix (group A) (*P:* A–B = 0.000, A–C = 0.000), with no significant difference between the AT LISA (group B) and the Symfony (group C) (*P* = 0.188). However, UIVA at 60 cm was significantly better in the Panoptix (group A) and the Symfony (group C) compared to the AT LISA (group B) (*P:* A–B = 0.000, B–C = 0.000), with no significant difference between Panoptix (group A) and the Symfony (group C) (*P* = 0.095). Regarding DCNVA, statistically significant lower values were recorded in the Symfony (group C) when compared to the other 2 groups (*P* = 0.000, 0.000 respectively). The mean postoperative SE and the mean postoperative cylinder were similar among the three types of IOLs (Table 2).

**Table 2:** Comparison of postoperative visual outcomes between the three groups.

Parameter	PanOptix (A)	AT LISA tri (B)	Symphony (C)	*P
UDVA				
Median	0.09	0.08	0.07	0.23
Min, Max	(−0.16, 0.28)	(−0.14, 0.24)	(−0.12, 0.26)	
Q1, Q3	(0.12, 0.20)	(0.00, 0.15)	(0.00, 0.17)	
CDVA				
Median	−0.06	−0.08	−0.1	0.3
Min, Max	(−0.23, 0.02)	(−0.2, 0.00)	(−0.21, 0.03)	
Q1, Q3	(−0.12, 0.00)	(−0.12, 0.00)	(−0.1, 0.00)	
UIVA (80 cm)				0.000
Median	0.19	0.04	0.05	A–B: 0.000
Min, Max	(0.00, 0.6)	(−0.2, 0.15)	(−0.17, 0.3)	A–C: 0.000
Q1, Q3	(0.08, 0.26)	(0.00–0.00)	(0.01–0.1)	B–C: 0.188
UIVA (60 cm)				0.000
Median	0.00	0.15	0.01	A–B: 0.000
Min, Max	(−0.06, 0.11)	(−0.06, 0.5)	(−0.04, 0.13)	A–C: 0.095
Q1, Q3	(−0.03, −0.03)	(0.02, 0.31)	(−0.01, 0.04)	B–C: 0.000
UNVA (40 cm)				0.000
Median	0.18	0.17	0.3	A–B: 0.3
Min, Max	(0.08, 0.32)	(0.06, 0.28)	(0.1, 0.6)	A–C: 0.000
Q1, Q3	(0.12, 0.24)	(0.1, 0.23)	(0.2, 0.41)	B–C: 0.000
DCNVA (40 cm)				0.000
Median	0.00	−0.01	0.2	A–B: 0.24
Min, Max	(0.08, −0.14)	(0.06, −0.16)	(0.00, 0.3)	A–C: 0.000
Q1, Q3	(0.06, −0.04)	(0.02, −0.05)	(0.17, 0.21)	B–C: 0.000
SE (D)				
Mean	−0.31 ± 0.37	−0.23 ± 0.42	−0.19 ± 0.32	0.3
Range	(−0.9 to 0.35)	(−0.96 to 0.59)	(−0.75 to 0.35)	
Cylinder (D)				
Mean	−0.08 ± 0.3	±0.24	−0.09 ± 0.32	0.26
Range	(−0.63 to 0.44)	(−0.57 to 0.67)	(−0.6 to 0.53)	

*P Kruskal–Wallis test among the three groups followed by Dunn–Bonferroni post hoc analysis if *P* < 0.05.

### Binocular distance-corrected defocus curve

**Fig. 2:**
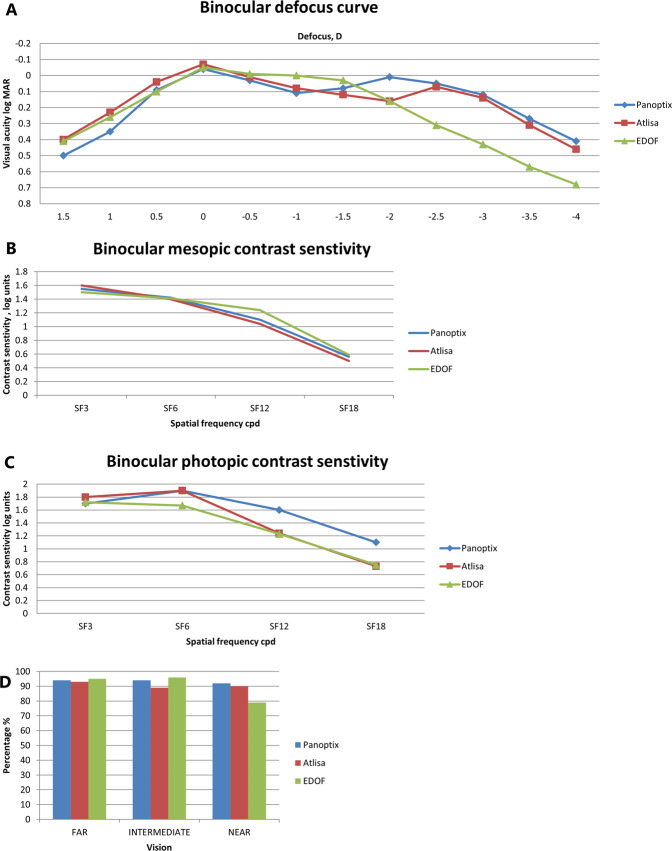
Binocular defocus curves, photopic, scotopic contrast sensitivities, and spectacle independence of Panoptix IOL, AT LISA tri IOL and Symphony IOL. **A** Binocular defocus curves 6 months after implantation of PanOptix IOL, the AT LISA IOL and Tecnis Symfony IOL. D diopter, logMAR logarithm of the minimum angle of resolution). **B** Binocular scotopic contrast sensitivity without glare 6 months after implantation of PanOptix IOL, the AT LISA IOL and Tecnis Symfony IOL (Cpd cycle per degree). **C** Binocular photopic contrast sensitivity without glare 6 months after implantation of PanOptix IOL, the AT LISA IOL and Tecnis Symfony IOL (Cpd cycle per degree). **D** Percentage of spectacle independence for far, intermediate and near vision reported 6 months after implantation of PanOptix IOL, the AT LISA IOL and Tecnis Symfony IOL.

In the three groups, visual acuity of 0.3 logMAR or better was achieved with defocus levels from −2.5D (40 cm) to 0.0D (4.0 m). The best results for PanOptix group were obtained at defocus of 0.00 D and −2.00 D, simulating a distance of 4.0 meters (Far) and 50 cm (intermediate), with visual acuity of −0.04 logMAR and 0.01 logMAR respectively. For the AT LISA group, the best VA (−0.07 LogMAR) was obtained with a defocus of 0.00 D (4 m), then VA progressively decreased with negative defocus, however, a second peak of good VA was found at −2.5 D (40 cm) (0.07 LogMAR). The defocus curve for the Symfony IOL demonstrated the best VA (−0.05 LogMAR) at a defocus level of 0.00 D (Fig. 2A).

At a defocus of −2.50 D (40 cm), The near VA was similar in the PanOptix and AT LISA groups (0.05, 0.07 logMAR respectively), which were significantly better than the Symfony group (0.3 LogMAR) (*P* = 0.000). At a defocus of −2.0 D (50 cm), The PanOptix group had a statistically significantly better VA than both the AT LISA and The Symfony groups (LogMAR = 0.01, 0.17, 0.18 respectively) (*P* = 0.000). The worst intermediate VA was obtained at a defocus of −1.00 D (1 m) in the PanOptix group and −2.00 D (50 cm) in the AT LISA and symphony groups. Symfony IOL group had better VA than PanOptix and AT LISA groups with defocus of −1.0 D and −1.50 D.

### Contrast sensitivity

As demonstrated in Fig. 2B, C binocular scotopic and photopic contrast sensitivity outcomes were similar among the 3 groups at all spatial frequencies (*P* > 0.05).

### Photic phenomena

There were no statistically significant differences in the frequency, severity and degree of bother of photic phenomena when comparing the three studied groups; PanOptix and AT LISA and Symphony groups (Table 3).

**Table 3:** Frequency, severity and degree of bother of photic phenomena reported by IOL group.

		Panoptix (A) *n* = 26	AT Lisa (B) *n* = 27	Symphony (C) *n* = 26	*P
Frequency					
Glare	Never	12	14	9	A–B: 0.8
	Occasionally	8	9	6	A–C: 0.16
	Quiet often	4	3	8	B–C: 0.17
	Very often	2	1	3	
Halo	Never	6	7	10	A–B: 0.9
	Occasionally	4	4	6	A–C: 0.2
	Quiet often	12	11	5	B–C: 0.36
	Very often	4	5	5	
Starburst	Never	16	13	9	A–B: 0.74
	Occasionally	6	9	12	A–C: 0.16
	Quiet often	3	3	2	B–C: 0.67
	Very often	1	2	3	
Severity					
Glare	Not at all	14	13	11	A–B: 0.8
	Mild	6	9	9	A–C: 0.17
	Moderate	4	4	5	B–C: 0.16
	Severe	2	1	1	
Halo	Not at all	9	9	5	A–B: 0.9
	Mild	9	10	8	A–C: 0.46
	Moderate	6	7	10	B–C: 0.49
	Severe	2	1	3	
Starburst	Not at all	15	16	12	A–B: 0.9
	Mild	4	4	5	A–C: 0.35
	Moderate	6	5	6	B–C: 0.36
	Severe	1	2	3	
Degree of bother					
Glare	Not at all	15	14	9	A–B: 0.78
	Little	7	8	8	A–C: 0.17
	Quite	4	4	6	B–C: 0.48
	Very	–	1	3	
Halo	Not at all	13	12	11	A–B: 0.53
	Little	8	9	8	A–C: 0.97
	Quite	5	4	5	B–C: 0.53
	Very	–	2	2	
Starburst	Not at all	18	14	11	A–B: 0.88
	Little	6	6	6	A–C: 0.35
	Quite	2	5	6	B–C: 0.08
	Very	–	2	3	

^*^Chi square test.

### Spectacle independence

The majority of patients in the three groups reported spectacle independence for far, intermediate and near vision (Fig. 2D). However, higher percentage of patients in symphony group reported their need for reading glasses to improve their near vision.

## Discussion

The current study revealed an acceptable refractive outcome for the three IOLs, with a mean postoperative sphere and cylinder within ±1.0 DS, ±1.0 DC respectively. This goes in agreement with previous reports of the three IOLs [17–19]. Good UDVA and CDVA were achieved in the three groups, with no statistically significant difference among them. PanOptix and Symfony IOLs showed a statistically significant better intermediate VA at 60 cm than AT LISA IOL, while AT LISA IOL showed a statistically significant better intermediate VA at 80 cm than PanOptix and Symfony IOLs. Symfony IOL showed a statistically significant worse DCNVA compared to the other two groups. These results are consistent with previous studies [19–24].

Comparison of the visual performance of different presbyopia-correcting IOLs among different studies may be difficult due to different study designs, variable acuity charts utilized. Standardized defocus curve testing provides an accurate measure of the functional vision range of each IOL at variable distances. We found good functionality and acceptable levels of VA from near to far viewing in the three types of IOLs. The defocus curve obtained for PanOptix and AT LISA IOLs showed a “bifocal” defocus profile with two clear peaks of maximum vision. The best visual acuity was obtained at distance, followed by a slight drop of VA for intermediate vision, then a slight improvement for near vision. On the other hand, The Symfony IOL had the “smoothest” defocus curve profile, with a more progressive visual acuity decrease with increasing levels of defocus. Our results go in agreement with previous reports [19–24]. Ruiz-Mesa et al. [21] reported a comparable defocus curves pattern for both PanOptix and Symfony IOLs, for far and intermediate vision, but PanOptix achieved significantly better near visual acuities between −2.00 D and −4.00 D than Symfony. Similarly, Monaco et al. [22] reported that PanOptix IOL gave a statistically significantly better VA than Symfony IOL at defocus level of −1.5 D, and from −2.5 D to −4.0 D. Mencucci et al. [23] found that at 60 cm, PanOptix group had a better intermediate VA than the other two groups; while at 80 cm, Symfony group had significantly better intermediate VA than the other 2 IOLs. PanOptix group had the best near VA, followed by AT LISA; both had a significantly better near VA than Symfony group.

It has been previously questioned whether the splitting of incoming light into two or more foci worsen the contrast sensitivity compared with bifocal IOLs. It has been concluded that addition of a third focus is unlikely to decrease the quality of distance vision [25–27]. We could not find statistically significant differences in contrast sensitivity between IOLs at any spatial frequency under both photopic and scotopic conditions. Bohm et al. [5] reported similar binocular contrast sensitivity outcome under photopic conditions with PanOptix and AT LISA IOLs. Previous studies also found no difference in contrast sensitivity between PanOptix and Symphony IOLs under both scotopic and mesopic conditions [21, 26]. Moreover, Sudhir et al. found that the contrast sensitivity for the three types of IOLs was within normal range for age of the study population [20].

The main reason for patients’ disappointment after presbyopia-correcting IOLs implantation is photic phenomena, including halos, glares, and starbursts. In spite of high perception of these phenomena reported with these IOLs, they are usually non-bothersome and gradually decrease with time due to neuroadaptation [28]. It has been reported that trifocal IOLs have a less incidence of photic phenomena than bifocal IOLs [25]. Moreover, EDOF lenses with the echelette design provide an elongated range of focus rather than individual focal points, thus reducing photic phenomenon [11]. Therefore, they have been recommended for patients concerned with visual disturbances [12, 29], with the tradeoff for a worse near vision [27]. Our results suggest that this compromise in quality of vision is not necessary, as the visual disturbances reported in PanOptix and AT LISA groups were similar to those reported in Symfony group. The higher number of patients reporting bothersome visual phenomena was not statistically significant. Escandón-García et al. [24], found that PanOptix and Symfony IOLs induced similar light disturbance as measured with light distortion analyser. Other studies reported higher frequency with a greater degree of bother of photic phenomena with Symfony IOL than with PanOptix and AT LISA IOLs [21, 23, 24]. On the other hand, Lubiński et al. [27] reported lower incidence of glare and halo in the Symfony group compared to AT LISA IOL. They thought that Symfony IOL corrects corneal chromatic and spherical aberrations, thus creates a sharper light focus. On the contrary to the diffractive IOLs, the Symfony IOL lack the diffractive steps responsible for glare and halo.

We reported a relatively higher spectacle independence for the PanOptix and AT LISA IOLs relative to the Symfony IOL, which was consistent with previous studies [30, 31]. The need for reading glasses in the Symfony group is related to defective near vision provided by this lens as proved by defocus curve.

The study limitations include subjective evaluation of photic phenomena and quality of vision. Most IOL studies use variable questionnaires to subjectively evaluate visual outcomes, which render accurate estimation of the incidence of photic phenomena difficult. Additional studies using the aberrometry for accurate objective comparison of the visual outcomes of IOLs is necessary. However, we comprehensively evaluated other clinical outcomes, including defocus curve and contrast sensitivity. Another limitation is the short follow-up period. Longer follow-up periods are needed as the process of neuroadaptation might influence the perception of visual phenomena.

With the availability of many presbyopia-correcting IOLs, knowing the behavior of each IOL is essential for choice of the best IOL design compatible with patient’s needs and expectations, for achieving the best satisfaction. PanOptix IOL and AT LISA IOL would be a good choice for patients aiming for an optimum near vision, while Symfony IOL seems suitable for patients having the priority for good intermediate vision, such as electronic device frequent users (smartphones, computers and tablets). The compromise of quality of vision was similar between the three groups.

## Summary

### What was known before

- Different types of presbyopia-correcting IOLs had been used through last years with different results.

### What this study adds

- Knowing the behavior of each IOL is essential for choice of the best IOL design compatible with patient’s needs and expectations, for achieving the best satisfaction. PanOptix IOL and AT LISA IOL would be a good choice for patients aiming for an optimum near vision, while Symfony IOL seems suitable for patients having the priority for good intermediate vision, such as electronic device frequent users (smartphones, computers and tablets). The compromise of quality of vision was similar between the three groups.

## Data Availability

Data supporting the findings of this study are available from the corresponding author on request.
