# Editorial Expression of Concern: Visual performance following implantation of presbyopia correcting intraocular lenses

**DOI:** 10.1038/s41433-024-03526-y

**Published:** 2025-01-07

**Authors:** Magda A. Torky, Amgad El Nokrashy, Heba Metwally, Ameera G. Abdelhameed

**Affiliations:** 1 Department of Ophthalmology, Mansoura Ophthalmic Center, Faculty of Medicine, Mansoura University, Mansoura, Egypt; 2 Memorial Institute of Ophthalmic Research, Mansoura, Egypt

**Keywords:** Medical research, Health care

**Editorial Expression of Concern to:** *Eye* 10.1038/s41433-022-02188-y, published online 8 August 2022

The Editor-in-Chief would like to alert the readers that concerns have been raised regarding this article. Concerns include obtaining ethical approval before the start of this study, the number of participants involved in the study, and their consent to participate. Despite the efforts made to obtain an explanation and clarification from the authors regarding these concerns, it appeared that no documentary evidence could be made available by the authors to support their explanation.

All authors disagree with this Editorial Express of Concern.

